# Survey datasets on sick building syndrome: Causes and effects on selected public buildings in Lagos, Nigeria

**DOI:** 10.1016/j.dib.2018.08.182

**Published:** 2018-09-05

**Authors:** David Obinna Nduka, Babatunde Ogunbayo, Adekunle Ajao, Kunle Ogundipe, Benjamin Babalola

**Affiliations:** Department of Building Technology, Covenant University, Ota, Nigeria

## Abstract

This dataset focuses on the causes and effects of sick building syndrome among users of selected facilities in Lagos. A mixed research approach of field measurement and cross-sectional survey was adopted. Descriptive statistics were implemented on the data acquired and are reported on tables and figures. The significance of this data leverages on providing insight and consciousness of sick building syndrome to users and occupants of constructed facilities. The survey dataset when analyzed can show direction on physical quantities levels that can be experienced in public buildings in tropical region.

## Specifications table

**Table:** 

Subject area	*Building Maintenance*
More specific subject area	*Facilities Management and Construction Technology*
Type of data	*Table, text file and figure*
How data was acquired	*Field survey*
Data format	*Raw, filtered and analysed*
Experimental factors	*Purposive sampling of selected users and Field measurement*
Experimental features	*Structured questionnaire and use of instruments (Thermoigrometer and BK precision Light Meter)*
Data source location	*Lagos, Nigeria*
Data accessibility	*All the data are contained in this data article*

## Value of the data

• The dataset provided symptoms associated with sick building syndrome and can be adapted for studies in other facilities, hence relating the results to different building facilities.
• The data signposted the facilities users state of improvement over symptoms of sick building which can present a debate for further studies in the same or other climatic conditions.
• Understanding the physical properties like temperature, relative humidity and lighting levels compatible with human comfort in building can guide designers and construction professionals on materials and construction techniques appropriate for a particular climatic condition.
• The dataset can increase awareness on the negative impact of defects in buildings and the relationship with emergence of sick building on the built environment.

## 1 Data

**Fig. 1:**
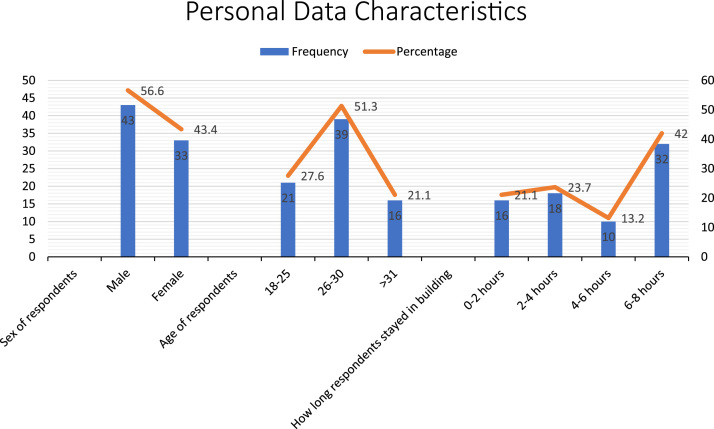
Summary of personal data of respondents.

**Fig. 2:**
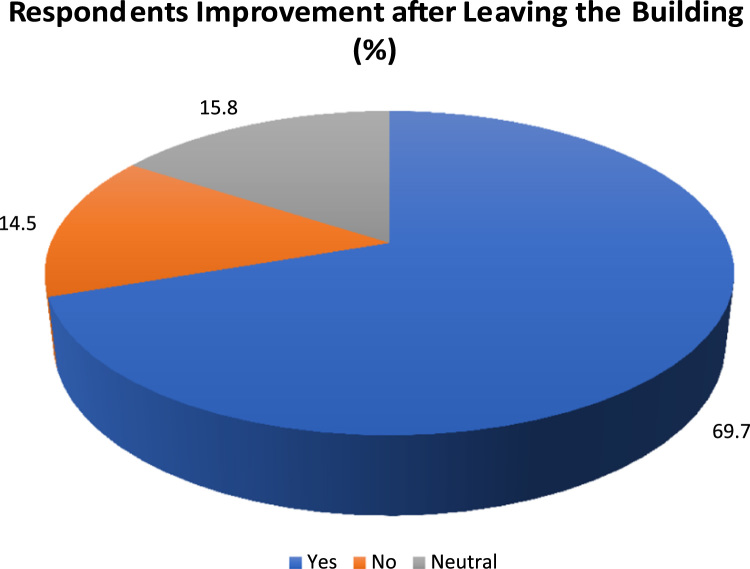
Respondent improvement after leaving the building.

This dataset explores the causes and effects of sick building syndrome on users in public facilities in University of Lagos, campus. In achieving the objectives of the dataset, opinions of 30 staff of three different banks and 46 users and worshippers in the university׳s worship centers in different locations on campus were sampled through structured questionnaire. Personal data characteristics of the respondents are shown and summarized in Fig. 1. Additionally, data were collected through field measurement using Thermoigrometer instrument for measuring temperature and relative humidity respectively while BK Precision Light meter instrument was used to measure lighting levels in the internal spaces. The analyzed data identified various symptoms linked to sick building syndrome in selected the facilities as contained in Table 1. Fig. 2 shows the facilities users state of improvement over the symptoms of sick building syndrome when not in the building. Further study of the data can offer understanding into the factors that affect the human comfort in the building and the consequences of defects in building as reflected in Tables 2 and 3.

**Table 1:** Sick building syndromes symptoms experienced in building.

**S/N**	**Symptoms**	**Yes (%)**	**No (%)**	**Neutral (%)**	**Ranking**
1	Sensitivity to odours	57.7	80.8	14.1	1
2	Sneezing	56.4	28.2	15.4	2
3	Coughing	53.8	35.9	10.3	3
4	Tiredness	52.6	35.9	7.7	4
5	Headache	47.4	48.7	3.8	5
6	Dizziness	38.5	52.6	9.0	6
7	A sensation of difficulty in breathing	36.5	55.5	8.0	7
8	Blocked or stuffy nose	34.6	57.7	7.7	8
9	Watery eyes	30.8	61.5	7.7	9
10	Running nose	26.9	65.4	7.7	10
11	Dry throat	26.9	60.3	12.8	10
11	Difficulty/poor concentration	17.9	71.8	10.3	12
12	Tightness of the chest	12.8	78.2	9.0	13
13	Dryness and irritation of the skin	11.5	80.8	7.7	14

**Table 2:** Factors that affect human comfort in buildings.

**Factors**	**Mean**	**Rank**
**Ventilation**		
Air intake sited away from source of contamination	1.67	36
Positioning of building with the wind direction towards source(s) of pollution	2.56	3
The use of air filters for the cooling system fitted correctly	1.99	24
Adequacy of windows(s) for ventilation of occupants	1.82	27
The arrangement of furniture׳s prevents blockage of air cooling system	2.50	4
Provision of inlet and extract vents in the rooms	2.37	5
Enduring satisfactory air circulation by air conditioning units	2.00	20
**Humidity**		
Relative humidity maintained between 40% and 60%	1.94	25
Provision of electricity within the building all the time	2.20	10
**Lighting**		
The use of specific luminaires to alleviate screen glare on visual display units (VDUs)	2.30	6
The use of task light lighting (table light) to illuminate the room	2.50	5
Ensuring regular planned maintenance system is in operation	2.10	16
Ceiling and walls regularly decorated	2.10	16
Lighting system regularly maintained	1.80	28
**Cleaning**		
The building fabrics are regularly cleaned including exterior windows	1.70	31
internal surfaces such as carpets, floors and furniture are regularly cleaned	1.70	31
Regularly damp dusting on all hard surfaces	1.70	31
Cleaning fluids and chemicals used correctly to manufacturers’ specification	2.10	16
Regular cleaning of the vents	2.00	22
Regular cleaning of the luminaires	1.80	28
Ventilation ducts inspected and cleaned as necessary	2.20	10
Filing cabinets regularly vacuumed	2.20	10
**Use of building**		
The original occupancy level is achieved	1.90	26
Ensuring non-pasting of posters or any other item on the walls	1.70	31
The use of bin regularly maintained	1.80	28
Building management		
Computerized building management systems are in place	2.20	10
The remote systems are avoided	2.30	6
The complaints procedures available to occupants when working in the environment is unsatisfactory	2.30	8
Glare (excess) light is avoided in office space	2.20	10
Provision of windows shades for natural ventilation are available	3.00	1
Obtaining natural day lighting for occupant comfort	2.00	20
**Contaminants**		
Regular refurbishment as part of maintenance	2.00	20
Placement of photocopies and printers in sealed rooms with their own extract system	2.30	6
Consultation with occupants on furnishings.	2.60	2

**Table 3:** Possible defects in building.

s/n	**Building component/element defects**	**Mean**	**Ranking**
**A**	**Roof (wooden member)**		
1	Poor strength and stability of the timber framing resulting in sagging and spreading of roofs	2.90	45
2	Decay (particularly trusses and facia)	3.20	4
**B**	**Roof covering (asbestos)**		
1	Broken roofing sheets	3.50	2
**C**	**Roof covering (Aluminium sheet or corrugated zinc)**		
1	Roof leakage	3.20	4
2	Corroded or worn out	3.10	22
3	Leaking rain water gutter	3.10	22
4	Faulty roof drainage	3.10	22
**D**	**Sanitary fittings and appliance (Plumbing)**		
1	Septic tank full	3.20	4
2	Inefficient flushing of WC	3.00	34
3	Blocked trap of sanitary appliance	3.00	34
4	Leaking pipes	3.00	34
5	Faulty water taps	3.00	34
6	Worn out drainage board	3.00	34
7	Loose bracket holding pipes to walls	3.10	22
**E**	**Electrical**		
1	Broken switches and sockets	3.00	34
2	Worn out electrical insulated copper wires	3.10	22
3	Loose wall brackets	3.00	34
4	Cutting off electrical supply	3.20	4
5	Damage to luminaires by vandals	3.20	4
6	Loose arrangement of wires	3.20	4
**F**	**Staircases**		
1	Nosing, cracked or missing	3.10	22
2	Worn out nosing, treads, balusters, handrails, loose newels post	3.10	22
3	Handrail loose and baluster loose in their bases	3.10	22
4	Blocked rain water, gutter and drainage	3.20	4
**G**	**Walls (Sandcrete blocks)**		
1	Settlement cracks	3.20	4
2	Bulging and buckling (external walls only)	3.30	3
3	Dampness of walls	3.00	34
4	General weathering/erosion of wall surface	3.10	22
5	Atmospheric impurities	3.20	4
**H**	**Floors/Finishes**		
1	Spalling (with reinforcement exposed)	3.20	4
2	Movement cracks	3.20	4
3	Worn out screed/finish	3.20	4
4	Dirty terrazzo/granolithic	3.20	4
5	Worn out tiles, ceramic, PVC, clay quarry tiles, marbles, wood blocks	3.20	4
**J**	**Windows/door joinery**		
1	Decayed frames	3.20	4
2	Sticking of frames	3.20	4
3	Broken glazing	3.00	34
4	Screening noise in doors handles	3.60	1
5	Loose hinges	3.20	4
6	Loose louver blade	3.20	4
**K**	**Wall finishes (Paint)**		
1	Peeling	3.00	34
2	Chipping or flaking	3.10	22
3	chalking	3.10	22

## 2 Experimental design, materials and methods

The dataset adopted cross-sectional survey design and physical measurement methods. The data purposively sampled 100 respondents who were users and worshippers in the church and mosque and staff of three commercial banks within the University of Lagos, Akoka campus. The sample frame consists of 76 valid questionnaires comprising 30 bank staffers and 46 worshipers. Recent studies [1], [2], [3], [4], [5], [6], [7], [8], [9], [10], [11], [12], [13] have documented the negative effects of sick building syndrome on human health across climes. The survey instrument was administered by hand and consists of four parts. Objective assessment on three physical quantities: temperature, relative humidity and lighting levels were undertaken and presented in Table 4. Temperature and relative humidity were measured using Thermoigrometer instruments while BK Precision Light meter instrument was used in measuring the internal space lighting levels respectively. The temperature and relative humidity readings were taken during the day at 2 h intervals in the month of September in the selected facilities. The lighting levels in the internal spaces of worship centers only were measured in the daytime at 3 m intervals. The data collected were coded and keyed into the Statistical Package for Social Sciences (SPSS) IBM v.21 for analysis. Descriptive statistical tools such as frequency, percentage, mean and ranking were used to present the data.

**Table 4:** Physical quantities measurement.

**Facilities**	**Physical quantities**		
	**Lighting level (Lux)**	**Temperature (°C)**	**Relative humidity (%)**
**Worship centre A**	1280	30.5	50
	202	30.5	50
	183	30.5	50
	219	30.5	50
	750	30.5	50
	400	30.5	50
**Mean**	**400**	**30.5**	**50**
**Worship centre B**	295	30	60
	370	29	58
	295	29.5	59
	272	29.5	54
	530	28.5	58
	565	28	60
	274	39	59
	311	30	58
	910	30	60
	813	29.5	59
**Mean**	**464 lx**	**29 °C**	**59%**
**Bank A**		29	50
		28.5	49
		28	49.5
		28.5	49
		28	50
**Mean**		**27 °C**	**49%**
**Bank B**		30	49
		29	50
		29.5	57
		30	50
		28	50
**Mean**		**28 °C**	**50%**
**Bank C**		28	48
		28.5	49
		29	48.5
		28	48
**Mean**		**25 °C**	**48%**
