# Supplementary material for: Survey datasets on sick building syndrome: Causes and effects on selected public buildings in Lagos, Nigeria
Source: Data Brief. 2018 Sep 5;20:1340–6. doi: 10.1016/j.dib.2018.08.182 (PMC6146451; doi:10.1016/j.dib.2018.08.182)
Supplement: Supplementary file 1 — Supplementary material [file mmc1.docx]

Nduka David Obinna

Department of Building Technology

Covenant University, Ota, Nigeria

28^th^ July, 2018

The Editor

Data in brief

Elsevier

Dear sir/ma,

**CONFLICT OF NO INTEREST DECLARATION**

I hereby declare that there is no conflict of interest on this manuscript “Survey dataset on sick building syndrome: causes and effects on selected public buildings in Lagos, Nigeria”. This manuscript is a direct submission to Data in brief.

I wish to confirm that there are no known conflicts of interest associated with this publication and there has been no significant support for this work that could have influence its outcome.

I confirm that we have given due consideration to the protection of intellectual property associated with this work and that there are no impediments to publication, including the timing of publication, with respect to intellectual property. In so doing we confirm that we have followed the regulations of our institutions concerning intellectual property.

Thank you


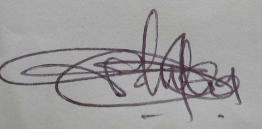


David O. Nduka
